# Osteopathic Manipulation as a Method of Cortisol Modification: A Systematic Review

**DOI:** 10.7759/cureus.36854

**Published:** 2023-03-29

**Authors:** Dylan Thibaut, Valentine Santarlas, Joseph Hoppes, Alejandra Vásquez-Castillo, Alexa Morrow, Eddie Oviedo, James Toldi

**Affiliations:** 1 Osteopathic Medicine, Lake Erie College of Osteopathic Medicine, Bradenton, USA; 2 Sports Medicine, Lake Erie College of Osteopathic Medicine, Bradenton, USA

**Keywords:** serum cortisol studies, salivary cortisol studies, osteopathic manipulative medicine, osteopathic manipulative treatment, doctor of osteopathic medicine, omm, osteopathic manipulation, sham, cortisol, omt

## Abstract

The effectiveness of osteopathic manipulative treatment (OMT) in the modification of various hormones has been studied; however, there is still a need for quantitative measurements to determine how large of an influence exists. The goal of this meta-analysis is to investigate the implications OMT has on cortisol levels. A systematic literature search restricted to English was performed from October 2022 to November 2022 using Google Scholar, OSTMED.DR, and PubMed and included articles from 2000 onward. Articles were excluded if they did not include a measurement for the control group in their study. We identified 4120 studies for potential inclusion. Of these, a total of four studies met the inclusion criteria, with a total of 135 participants (N= 68 OMT, N= 67 control). Out of the 135 participants, 126 participants (N= 62 OMT, N= 64 control) made up the salivary cortisol studies, and the remaining nine participants made up the serum cortisol studies (OMT N= 6, control N= 3). The National Institutes of Health (NIH) bias assessment tool was utilized to measure the risk of bias. Standard mean differences were calculated for effect size. A mean difference in cortisol of 0.10μg/dl (-10μg/dl, 95%CI -0.15, -0.04) was found when comparing all pre- versus post-cortisol levels with OMT versus sham control groups. OMT demonstrated a 0.10μg/dl larger decrease in cortisol than sham control treatments. The standard mean difference was found to be -0.46 (95%CI -1.40, 0.48) making this finding a medium effect size without significance. Heterogeneity for the salivary analysis measured by I^2^ was 0% indicating no significant heterogeneity across studies. When serum cortisol was included, heterogeneity stayed at 0%. A larger number of high-quality studies, especially those specific to serum cortisol, are recommended, to elucidate the relationship between OMT and cortisol. This research suggests OMT reduces cortisol more than sham treatment before versus after OMT treatment, and though the change is small when comparing after one treatment, it may have clinical usage if multiple OMT sessions are performed.

## Introduction and background

Osteopathic manipulative treatment (OMT) serves as a modality to potentially assist in the treatment of various illnesses. Through manipulation treatment, osteopathic physicians offer an additional method by which healing can be promoted in patients. While these treatments may have effects on various biomarkers, the exact changes are unknown. Bodily manipulation is well established in-vitro, with fibroblasts indicating changes in what is released, how cells change, and bodily interconnection [1-4]. It follows from these studies that manipulation would similarly show these changes in patients. From interleukins to white blood cells to hormones, OMT has shown promise for causing bodily changes in research [5-7].

The common problem with OMT research is that studies often have low power, low sample size, or questionable experimental design [8]. To better analyze the effects of osteopathic medical research with these limitations, meta-analysis serves as an approach to a more evidence-based method to find overall effects [9,10]. While individual studies can be inconclusive or have problems with following up on patients receiving treatment, the effect of these problems may be minimized through the inclusion of other studies with small samples. Beyond these problems with limited samples, there may be a challenge in getting a consistent measurement from multiple participants. This is especially true of biomarkers that may be overlooked or be too small in concentration to be actively examined.

For something like cortisol, which often is only in concentrations of micrograms per deciliter in the blood or salivary secretions, the minute differences are one such thing that may be overlooked [11]. Cortisol serves a vital function in stress response, playing a role in adapting to change in everything from vitals to overall wellness [12-14]. Cortisol's effect on the sympathetic nervous system has an impact on many systems, such as cardiovascular (increased heart rate (HR) and blood pressure (BP)), gastrointestinal (inhibited digestion), and stimulation of adrenal glands (increased norepinephrine and epinephrine production). OMT’s potential to modify breathing and cardiac variability, especially if it has some effect on cortisol in a meaningful way, may have potential application for a multitude of conditions [15]. OMT has shown some potential in the regulation of cortisol, modifying stress responses, and being part of treatment to affect everything from vitals to the stress response [16,17].

With cortisol established as a necessary component of the stress response, its potential use in treatment modalities, and OMT possibly being able to affect cortisol concentration, an exploration of OMT and cortisol via meta-analysis is necessary. The objective of this study is to combine study results as a systematic review to find the overall change of cortisol in OMT versus sham treatments. Through this finding, the hope is to explore if there is a difference found in OMT that can have potential use in treatment.

## Review

Materials and methods

Studies were included based on specific inclusion criteria. To be included, a study needed to have pre- and post-treatment data, a defined control group, standard deviation as well as effect size and mean (or adequate data to calculate them), sample size for OMT and sham groups, cortisol (measured as salivary cortisol or serum cortisol), and a specified and recognized OMT treatment. Additionally, cortisol values were measured in μg/dL units in this nlysis, so all included studies needed data that was convertible to these units. Exclusion criteria were the following: studies that were not written in English, studies published prior to 2000, and studies that used alternative means to measure cortisol other than salivary or serum cortisol .

As for database search, Google Scholar, Osteopathic Medical Digital Repository (OSTMED.DR®), and PubMed were used. PubMed and OSTMED.DR had limited search results, and all search results found on them were also found on Google Scholar. The search term used in the search was “osteopathic” AND “cortisol.” After the initial article results, articles were eliminated first based on the language. This was done by using each database's option to only find English-only articles. After this, articles were excluded based on the other inclusion and exclusion criteria.

The search was divided among three researchers. Article search results were compiled together onto a shared document and each of the three researchers was given part of the search results to go through. Data gathered by the researchers were compiled into a shared document. If a researcher disputed an article for analysis, the article was given to the other two researchers doing the search to analyze. If the researchers still disputed whether the article should be included, the article was presented to the principal investigator of the project for a final say. Lastly, articles that did not include a control group measurement in their study were excluded from the final group. An example of this is Henderson et al., where placebo group changes in salivary cortisol were reported in terms of percent change [6]. Percentage change does not provide a quantifiable number that can be used in the statistical analysis.

Data collected included control and OMT group sample size, standard deviation, mean difference (or pre- and post-mean) for control and OMT groups, and OMT technique comparison. If multiple time points were given, the time points measuring closest to before and after the treatment was used. Risk of bias was measured using the National Institutes of Health (NIH) bias assessment tool. For effect size measurement, the standard mean difference was calculated [18].

Statistical analysis and sensitivity testing were performed using RevMan software [19]. Heterogeneity was measured using I^2^, in which a value above 50% was considered significant heterogeneity. Sensitivity testing was used to determine the highest contributors to heterogeneity for the results. Subgroup analysis was conducted for salivary cortisol as well as serum cortisol, with a total comparison conducted as well. This subgroup analysis helped in determining if an overall pattern was apparent in cortisol changes or if one measurement of cortisol was irrelevant to cortisol level change. As for bias assessment, studies were rated as good, fair, or poor. Analysis was conducted including and not including studies found to be poor in order to better see if there was an effect if they were excluded; 95% confidence intervals were generated for the mean difference and standard mean difference effect size measurement.

Results

**Figure 1:**
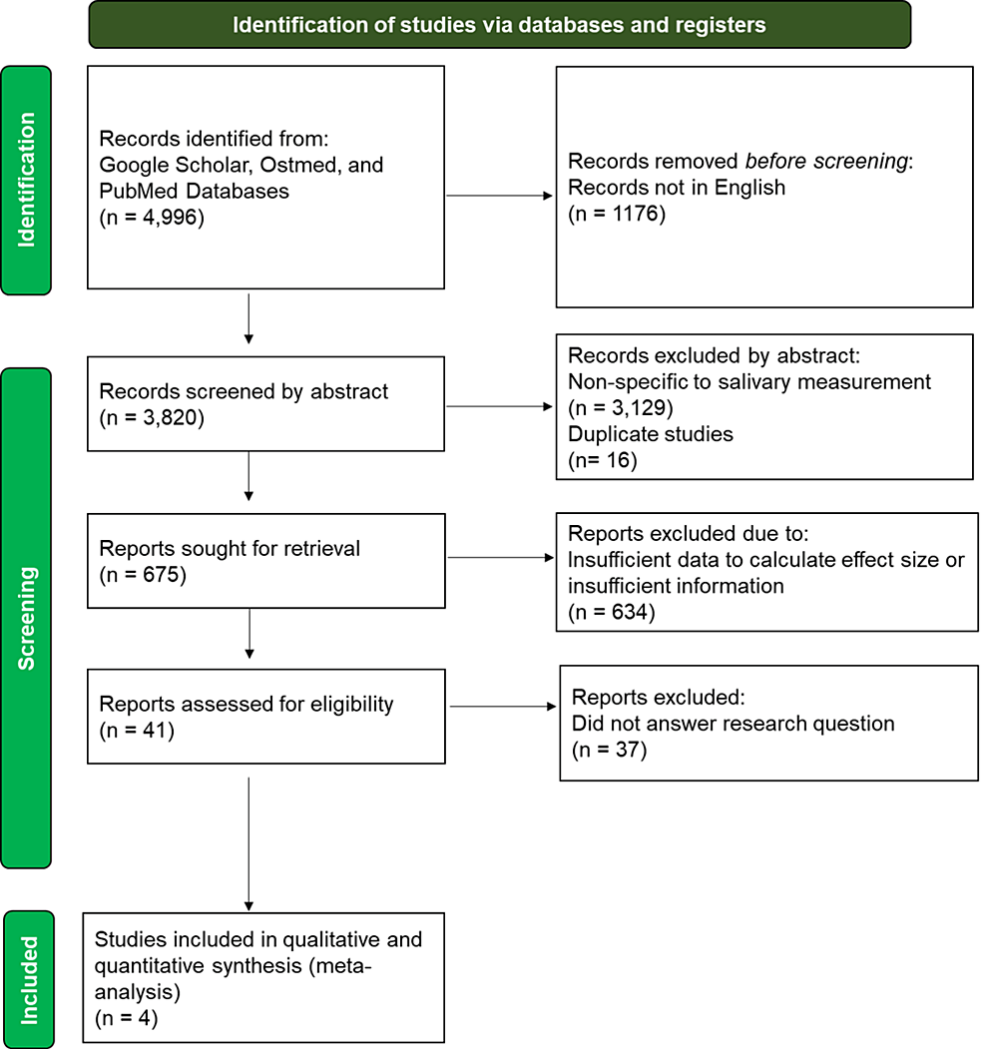
PRISMA Flowchart PRISMA: Preferred Reporting Items for Systematic Reviews and Meta-Analyses

A total of four studies met the inclusion criteria for this study with a total of 135 participants (N= 68 OMT, N= 67 control). Out of this number, 126 participants (N= 62 OMT, N= 64 control) made up the salivary cortisol studies, and 9 participants made up the serum cortisol studies (OMT N= 6, control N= 3) [20-23]. Controls and participants were not separated based on gender or age. Figure 1 shows the PRISMA flow diagram for the search strategy used for the meta-analysis. Values used for the meta-analysis used μg/dl to have a consistent measurement unit for comparison prior to analysis. Abraham et al.'s study was the only one to meet the inclusion criteria as a serum cortisol study rather than salivary cortisol [20].

Bias assessment of included studies is summarized in Table 1, with each number on the table indicating the numbered question on the NIH bias assessment tool [18]. These 14 parameters, in order of the assessment tool, were: (i) description of study type, (ii) randomization, (iii) allocation of treatment, (iv) blindness of participants to treatment group status, (v) blindness of participants to researchers, (vi) subject similarities at baseline, (vii) dropout rate at the end versus number allocated to treat, (viii) differental drop-out rate, (ix) adherence, (x) avoidance of other interventions, (xi) outcomes assessment approach, (xii) power calculation, (xiii) pre-specified outcomes, and (xiv) intention to treat [18]. Three classifications were used to grade the bias interpretation: poor if five or more of the 14 parameters were not found, fair if the study had three or four missing parameters, and good if there were less than three. These classifications were used in order to have a more consistent standard for what is considered biased, as it is normally a subjective judgement based on the totality of results. Bias assessment found that Vreede's was the strongest of the four included studies and the studies by Mieczikowski et al. as well as Fornari were fair [21-23]. Only one study was found to be poor, that of Abraham et al., due to its low sample size as well as the lack of several available bias assessment measures being reported in the publication [20].

**Table 1:** Bias Assessment NIH Bias Assessment Tool was used [18] CD: could not determine; NR: not recorded; Y: yes; N: no 1: description of study type; 2: randomization; 3: allocation of treatment; 4: blindness of participants to treatment group status; 5: blindness of participants to researchers; 6: subject similarities at baseline; 7: dropout rate at the end versus number allocated to treat; 8; differential drop-out rate; 9: adherence; 10:  avoidance of other interventions; 11: outcomes assessment approach; 12: power calculation; 13: pre-specified outcomes, 14 intention to treat

Study	Overall	1	2	3	4	5	6	7	8	9	10	11	12	13	14
Abraham et al., 2021 [20]	Poor	Y	Y	Y	N	Y	Y	N	N	Y	Y	Y	N	N	Y
Mieczikowski et al., 2020 [22]	Fair	CD	CD	CD	N	Y	Y	Y	Y	Y	Y	Y	Y	Y	Y
Vreede, 2020 [21]	Good	Y	Y	Y	Y	Y	Y	Y	Y	Y	Y	Y	N	Y	Y
Fornari, 2017 [23]	Fair	Y	Y	NR	N	Y	Y	Y	Y	Y	Y	Y	N	Y	Y

**Figure 2:**
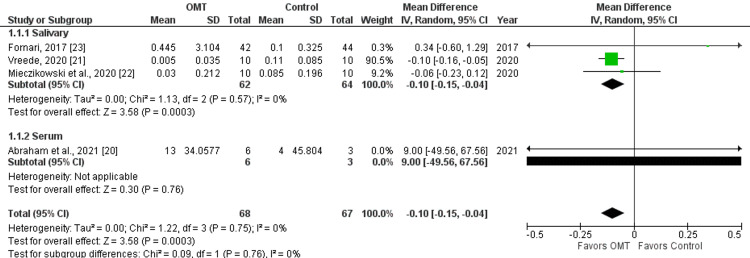
Forest Plot Analysis OMT: osteopathic manipulative treatment

Meta-analysis findings are summarized in Table 2 and Figure 2. A mean difference in cortisol of 0.10 μg/dl (-0.10 μg/dl, 95%CI -0.15, -0.04) was found when comparing all pre versus post-cortisol levels with OMT versus control groups. OMT demonstrated a 0.10 μg/dl larger decrease in cortisol than sham control treatments. The standard mean difference was found to be -0.46 (95%CI -1.40, 0.48) making this finding a medium effect size without significance. Heterogeneity for salivary analysis measured by I2 was 0%, indicating no significant heterogeneity across studies. When serum cortisol was included, heterogeneity stayed at I2 =0%. Not that cortisol was measured before versus after treatment without specific time of day consideration; despite the time of day influencing cortisol level, the focus only on the change before versus after treatment made the variable starting cortisol not a factor in results. Sensitivity analysis including versus excluding this poor study did not greatly affect results, as seen in Figure 2.

**Table 2:** Meta-Analysis Findings SMD: standard mean difference

Group	Case (N)	Control (N)	Δ Mean [95% CI]	SMD [95% CI]	I²
Salivary	62	64	-0.10 [-0.15, -0.04]	-0.46 [-1.40, 0.48]	0%
Serum	6	3	9.00 [-49.56, 67.56]	0.21 [-1.18, 1.60]	NA
Both	68	67	-0.10 [-0.15, -0.04]	-0.46 [-1.40, 0.48]	0%

Discussion

The meta-analysis of four studies performed here found that osteopathic manipulative treatment overall led to a 0.10 μg/dl decrease (-0.10μg/dl, 95%CI -0.15, -0.04) in salivary cortisol compared to sham treatments. This change is a significant change with low heterogeneity (p= 0.003, I^2^= 0%) compared to sham treatment. Analysis of salivary cortisol alone versus a combined analysis of serum cortisol with salivary cortisol led to the same findings. The one study found to be poor on bias assessment ultimately did not make a substantial difference to findings; removing it led to near identical findings [20].

Further analysis of the results, however, shows that the effect size, as calculated by standard mean difference, suggests the change in cortisol determined through statistical analysis had low power. Considering the change in cortisol after a single treatment is such a low concentration, this finding is unsurprising. Additional treatments of OMT over time may show greater changes; there is reason to suggest a potential multiplicative effect of OMT if used multiple times. Previous research on OMT has found both acute and chronic differences due to OMT treatment [24-27]. A longer approach measuring multiple OMT treatments over time may be necessary to see if the effects are additive over time.

When comparing OMT effects on cortisol with chiropractic effects on cortisol, a similar decrease in cortisol is seen, albeit inconclusive [28-30]. Although chiropractic research was not included in the analysis of this study, future research combining it with OMT may help increase the studies available to compare hands-on treatment on cortisol. Other avenues to explore include whether individual osteopathic techniques have an advantage of cortisol modification compared to other techniques; while this study combined any OMT treatment under one umbrella, it may be possible that less effective treatments may have been combined with more effective treatments during the analysis. With more studies in the future specific to treatments, a better idea of the effect of OMT on cortisol may be found. More focus must be put into how other vitals change or affect measurements, how the time of day may influence cortisol changes, and how the type of OMT performed influences results. Additionally, a Likert scale assessing the perception of stress can further connect perceived versus found changes.

High cortisol also shows a correlation with cognitive impairment in dementia, cardiovascular risk, complications in the elderly, and problems with depression [31-34]. In all these circumstances, treatment can be complex, with medication treatment alone not necessarily treating the condition. Additionally, treatments often do not address cortisol directly, while OMT potentially can. During the coronavirus disease 2019 (COVID-19) pandemic, there has been evidence supporting high cortisol as a marker for worse outcomes in COVID-19 patients [35]. OMT can simultaneously address some of the complications of COVID-19 while additionally modifying cortisol. In conditions from prostate cancer to obesity, to exercise-induced amenorrhea, high cortisol is a factor with the potential to be modified with OMT [36-38]. With OMT serving as a much less invasive way to lower cortisol in these conditions versus medication treatments and its safety as a treatment modality, it does not hurt to use OMT as an addition when there is a potential benefit to cortisol modification.

## Conclusions

In the future, this meta-analysis can be used as a guideline to pursue other patterns between biomarker levels and OMT. A low number of studies concerning the effects of OMT on serum levels of biomarkers prevents significant statistical power from being achieved. With more studies and conclusive findings, changes in biomarker levels after OMT can be viewed more frequently as an additional parameter when assessing the effectiveness of treatment. While encouraging, the inconsistent use of both serum and salivary measures for cortisol as well as the low effect size, pose limitations on the results of this study. A larger number of studies with consistent measuring protocols and larger sample sizes would fortify any correlations between OMT and changes in biomarker levels in future research. Nevertheless, the difference of 0.10 μg/dl lower serum cortisol levels compared to sham treatments may be additive with subsequent OMT and has the potential to provide benefit to patient care.
